# Assessment of Unintentional Acute Pesticide Poisoning (UAPP) Amongst Cotton Farmers in Tanzania

**DOI:** 10.3390/toxics13040300

**Published:** 2025-04-11

**Authors:** Jones Ackson Kapeleka, Aiwerasia Vera Ngowi, Silvani Mng’anya, Sheila E. Willis, Joey P. Salmon, Keith F. Tyrell, Stephanie Williamson, Michael Eddleston, Alexander M. Stuart

**Affiliations:** 1 Tanzania Plant Health and Pesticides Authority (TPHPA), Arusha P.O. Box 3024, Tanzania; jak78tz@gmail.com; 2 Department of Environmental and Occupational Health, Muhimbili University of Health and Allied Sciences, Dar es Salaam P.O. Box 65001, Tanzania; vera.ngowi@gmail.com; 3 AGENDA for Environment and Responsible Development, Dar es Salaam P.O. Box 77266, Tanzania; semnganya@gmail.com; 4 Pesticide Action Network UK, Brighthelm Centre, Brighton BN1 1YD, UK; sheila@pan-uk.org (S.E.W.); joey@pan-uk.org (J.P.S.); ktyrell@ed.ac.uk (K.F.T.); sofojowo@yahoo.co.uk (S.W.); 5 Centre for Pesticide Suicide Prevention, University of Edinburgh, Edinburgh EH16 4TJ, UK; m.eddleston@ed.ac.uk

**Keywords:** alternatives, chlorpyrifos, cotton farmers, cypermethrin, highly hazardous pesticides, lambda cyhalothrin, personal protective equipment (PPE), pesticide exposure, profenofos

## Abstract

Pesticide poisoning is widely accepted as a major public health problem in low- and middle-income countries, especially in East Africa. However, a very high proportion of unintentional poisonings are either unreported or misreported. To help fill this knowledge gap, we investigated the scale of unintentional acute pesticide poisoning (UAPP) in cotton-growing communities in Tanzania by conducting a cross-sectional survey of 1074 cotton farmers or farm workers. The findings from this study revealed a high incidence of self-reported negative health effects following occupational pesticide exposure, with 48% of respondents experiencing symptoms of UAPP within the previous 12 months. Furthermore, the substantial proportion of UAPP incidents were associated with a few highly hazardous pesticides (HHPs), particularly profenofos, lambda cyhalothrin, and co-formulations with both chlorpyrifos and cypermethrin. Of those reporting UAPP, only 6% sought medical treatment in a formal healthcare setting. The findings from this study clearly indicates an urgent need for improved pesticide regulation, the scale up of community surveillance, and the promotion of less hazardous alternatives to the use of HHPs. We provide policy recommendations and identify alternatives to HHPs for cotton pest management.

## 1. Introduction

Poisoning caused by pesticides is widely accepted as a major public health problem in low- and middle-income countries (LMICs) [1,2,3,4], yet the true scale of the problem is unknown. It has been estimated that there are 28-385 million cases of acute pesticide poisoning amongst agricultural workers per year [3,5]. In 2005–2006, a survey of 6300 smallholder farmers across 24 countries from around the world recorded the highest levels of UAPP in African countries, including Tanzania [6]. In a more recent survey of 353 conventional cotton growers in Burkina Faso, an astonishing 99.72% of respondents reported experiencing UAPP at least once since they started using pesticides [7].

In addition to unintentional exposure, pesticide poisoning via deliberate self-harm is a significant problem in LMICs, with one in five global suicides estimated to be due to intentional self-poisoning with pesticides [8]. Furthermore, numerous epidemiological studies have found associations between occupational pesticide exposure and chronic illnesses, such as cancers, neurological illnesses, and reproductive and developmental disorders [9,10,11]. These are much more difficult to quantify. However, a high incidence of UAPP indicates widespread exposure to pesticides and suggests that chronic health problems due to pesticide exposure may also be prevalent.

Aside from the direct effects on health, pesticide poisoning places a heavy financial burden on LMICs (as well as in HICs) due to the hidden costs, such as medical costs and related burdens on health services and society [12]. A UNEP study of smallholder farming communities in 37 sub-Saharan countries estimated that the health costs of pesticide poisoning—defined as lost work days, outpatient medical treatment, and inpatient hospitalisation—amounted to USD 4.4 billion in 2005 [13]. Furthermore, regular episodes of mild- or medium-level health effects amongst farming households that lead to lost work days can have potential negative impacts on agricultural production [14,15].

Estimates of UAPP and related economic costs are likely to be significantly underestimated in LMICs. Several studies have shown that a very high proportion of unintentional poisonings are either unreported or misreported [16,17,18,19]. The estimates of pesticide poisonings are typically based on official data collected by medical professionals, and often from hospital records only. However, only a small portion of the most serious cases are seen by medical professionals. In addition, many reviews of LMIC hospital data are ad hoc, and many hospitalised cases are often missing from national records [16,20].

Where pesticide poisoning monitoring systems do exist, the range of data collected is often limited and of little value to policymakers. For example, in Tanzania, the national health information management system has only one category for all types of poisoning, and important details on the type of pesticide, the conditions of exposure, and the severity of the symptoms are not systematically collected [20]. This same DHIS2 health information management system is used in 67 LMICs [21]. Unless these countries have established additional systems for the systematic collection of data on UAPP, they will also lack essential information on this topic.

The lack of data on pesticide poisoning hinders the development of appropriate policy measures and the allocation of adequate resources or political will to tackle it [15,18]. Without information on which pesticides are involved in poisoning, or which practices lead to hazardous levels of exposure, interventions are unlikely to be targeted effectively.

To complement clinical-based surveillance systems, field surveys have emerged as valuable tools to gather information on UAPP. Such surveys collect self-reported data directly from exposed communities and focus on acute pesticide poisoning, often revealing a high level of poisoning in the communities studied [22,23,24,25,26,27].

A number of approaches for field surveillance exist. These range from household interviews with questionnaires to open semi-structured group discussions [28,29], e.g., Community Pesticide Action Monitoring (CPAM), a health monitoring tool developed by Pesticide Action Network Asia Pacific that employs Participatory Action Research techniques and questionnaires to build up a picture of the local conditions under which pesticides have been used. These can be complemented with passive sampling techniques to identify specific pesticides to which subjects have been exposed [30,31] and/or biological testing, such as measuring acetylcholinesterase levels in blood [32]. PAN UK has conducted many UAPP studies for over more than a decade. Retrospective, self-reporting of negative health effects following occupational pesticide exposure via survey questionnaires is central to the approach [5].

In Tanzania, cotton is a strategic crop. It represents about 10% of Tanzania’s agricultural GDP and directly sustains over 500,000 households, predominantly smallholder farmers [33]. Pesticides are used by 85.9% of cotton farmers [34]. However, in Tanzania, the magnitude of UAPP is severely underreported [19], with very few health surveillance studies documented. This study aims to help fill this knowledge gap by investigating the scale of self-reported UAPP in cotton-growing communities in Tanzania and identifying the pesticides involved, along with the main exposure routes and practices that lead to such poisoning. This information will be useful in informing policymakers and in the development of effective pesticide poisoning prevention initiatives.

## 2. Materials and Methods

This study used a mobile application developed by PAN UK, designed to support extension services and other relevant field workers to collect data directly from end users.

### 2.1. Study Area

**Figure 1:**
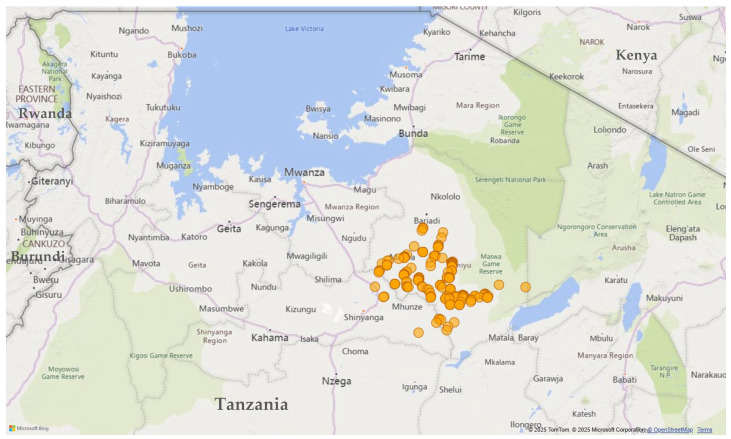
Map of survey location in Tanzania. Orange circles represent approximate interview locations.

This study was conducted in Simiyu region, which is one of the 31 administrative regions of Tanzania and the largest producer of cotton in the country (Figure 1). Simiyu region produces over 40% of the national cotton production. The region covers a land area of 25,212 km^2^ and is located along Latitude: −1°55′1.20″ S Longitude: 31°18′0.00″ E. Simiyu region is divided into five districts, namely, Bariadi, Busega, Itilima, Maswa, and Meatu, with the main economic activities including crop cultivation, mostly maize, sorghum, cotton, and cassava, as well as livestock rearing. Data were collected in November 2021 in Meatu and Maswa districts, where the agricultural economy of the area is dominated by cotton and cereal production.

### 2.2. Study Population, Sample Size Determination, and Sampling

Using a stratified random sampling technique, a list of villages in cotton production areas was identified by the District Agricultural Officers, and, from these lists, 14 villages were randomly selected from a total of 120 in Meatu, and 10 villages were randomly selected from a total of 109 in Maswa. However, we could not conduct interviews in 3 of these 24 villages, one in Maswa and two in Meatu, due to social–cultural events that were taking place. Thus, the survey was conducted in 9 villages in Maswa and 12 in Meatu. In each village, a minimum of 50 adult cotton farmers (including farm workers) were randomly selected for interviews, with a target proportion of 20% female. Only those who applied pesticides were interviewed. Those who were under 18 years old or did not apply pesticides on cotton farms were excluded from the survey.

### 2.3. Data Collection

Selected farmers were interviewed in person and individually using a structured questionnaire that was administered via the Tool for Monitoring Acute Pesticide Poisoning (T-MAPP) mobile phone application that was developed by PAN UK [5]. T-MAPP was developed in collaboration with Dr. Valentina Gallo, an epidemiologist at Queen Mary’s University, UK, to capture important information about the severity of UAPP incidents, as well as the type of pesticide and conditions of use. It was also developed to make data collection more efficient and to support trained enumerators to collect data directly from end users. To adapt the questionnaire for the local context, ‘drop lists’ of locally available pesticides, common crops, and names of crop pests were incorporated into the survey questionnaire in order to speed up the surveys and reduce inputting errors. The questionnaire was translated into Kiswahili and then pre-tested in the Kilosa district, Morogoro region, before making final revisions. Respondents were asked for their verbal consent before each interview, and the information collected was kept confidential and anonymous. Before asking for their consent to proceed with the questionnaire, each participant was informed about the purpose of the survey and the anonymity of data collection. All interviews were conducted in Kiswahili, a national language understood by all participants.

The questionnaire was divided into three sections. The first section concerned the respondents’ characteristics, the crops grown, the occurrence of major pests, and the conditions of pesticide use. The second section focused on incidents of UAPP experienced within the previous 12 months, and the name, formulation, and concentration of the pesticide that caused symptoms. Acute pesticide poisoning is variously defined when symptoms occur either within 24 h or within 48 h of exposure [2,35]. In this study, UAPP is defined as a symptom or health effect within 24 h of pesticide exposure.

If the respondent experienced such symptoms, follow-up questions about only the most recent incident involving a single product were asked in the third section of the questionnaire, following a further request for their consent. If a mixture of products was reported as the cause of exposure, the products were reported, and detailed questions on symptoms and health impacts were not asked. This section included closed questions regarding their symptoms. All respondents who reported UAPP were asked about symptoms relating to the nervous system and the hematopoietic system. For other organ systems, a triaging system was used to ask questions about the affected organ systems only, thereby avoiding asking detailed questions about unaffected organ systems. For each sign and symptom, sub-questions were added to determine its level of severity, derived from a classification tool developed for the International Programme on Chemical Safety [2]. Signs and symptoms that cannot be self-reported (e.g., massive haemolysis), are too open to misinterpretation if assessed by non-medical team staff (e.g., tinnitus or some cholinergic symptoms), or require medical instruments for their assessment (e.g., blood pressure tests) were excluded.

A total of 1123 participants were interviewed. The survey data were automatically uploaded to a PAN UK-managed database, which was exported to a csv file for data cleaning and analysis. During data cleaning, 49 reports were removed from the dataset based on incomplete records, giving a total of 1074 for analysis.

### 2.4. Data Analysis

Based upon the signs and symptoms reported, the severity of UAPP was categorised as either mild, moderate, or severe, in line with the severity scoring system proposed by Thundiyil et al. [2]. Data were processed and analysed using statistical programme SPSS version 22 (SPSS Inc., Chicago, IL, USA). To investigate factors influencing the occurrence of UAPP among farmers, binary logistic regression was conducted. The variables entered into the full model were age range, gender (male, female), farm size (1–5 ha, 5–15 ha, and >15 ha), occupation (farmer owner and not hired, farm owner and hired, and hired only), usage of PPE (0 PPE, 1 PPE item, 2–3 PPE items, and >3 PPE items), training in PPE use (yes, no), and the use of pesticides in original containers with original labels (yes, no). Chi-square or Fisher’s exact tests were used to find relationships between self-reported health effects and risk factors. A statistical analysis of the study was performed with a 95% confidence interval, and the different relationships observed were indicated with their associated *p*-value. Differences were considered statistically significant if *p* < 0.05.

## 3. Results

### 3.1. Social Demographic Characteristics of Respondents

A greater proportion of men were surveyed (82%) than women (18%). Respondent’s exact age was not recorded; instead, respondents provided their age within a range (Table 1). The main crops cultivated on family farms were cotton (98%), maize (37%), sunflower (5%), mung bean (4%), millet (2%), soya beans (2%), vegetables (2%), peanuts (1%), and potato (1%). The majority of respondents (97%) reported cotton requiring the most pesticide application by volume, followed by maize (2%) and other (1%). The most frequently reported pests targeted by pesticides were cotton stainers in 49% of reports, caterpillars (specific species not identified by respondent) in 36%, and American bollworm in 18%.

### 3.2. Conditions of Use of Pesticides in Cotton Production

**Figure 2:**
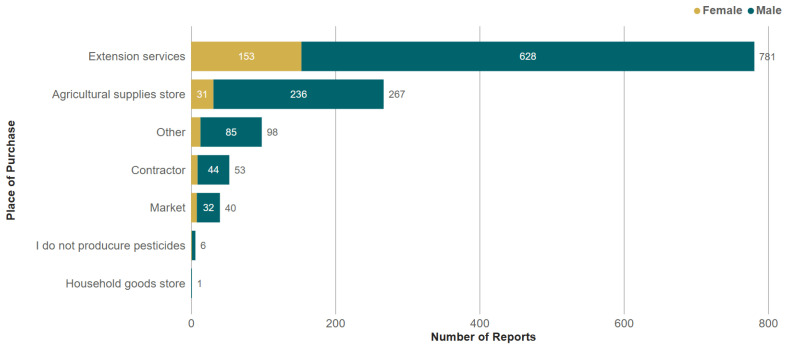
Frequency of reports for each place of purchase for pesticides. Respondents could report multiple options.

**Figure 3:**
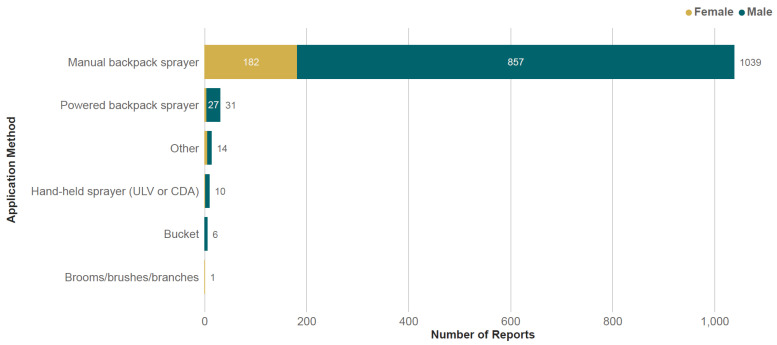
The frequency of pesticide application methods used by respondents. Respondents could report multiple options.

**Figure 4:**
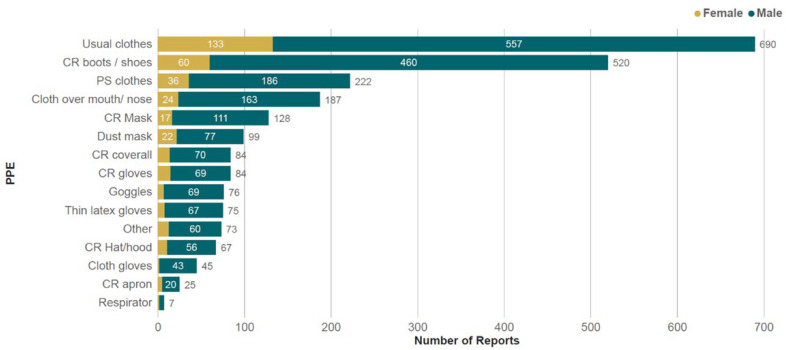
Percentage of respondents reporting items of clothing and PPE worn while handling pesticides, disaggregated by gender. Respondents could report multiple items. Key: CR: chemical resistant; PS: reserved for pesticide spraying.

The majority of respondents purchased pesticides from extension services (73%), followed by agricultural supply stores (25%), contractors (5%), or markets (4%) (Figure 2). The most frequently reported method of pesticide application was a manually operated backpack sprayer, followed by a powered backpack sprayer (Figure 3). About two-thirds of trained respondents (66%) received training in PPE in the year prior to the survey, 15% received training 1–2 years ago, and 7% received training 2–3 years ago. A slightly higher proportion of women received training in the previous year (79%) compared to men (65%). The most frequently reported clothing or PPE was ‘usual clothes’ (64%), followed by chemical-resistant boots/shoes (49%) and ‘ordinary clothes reserved for pesticide spraying’ (21%). The frequency of PPE use did not vary significantly between men and women (Figure 4).

### 3.3. Pesticide Poisoning

**Figure 5:**
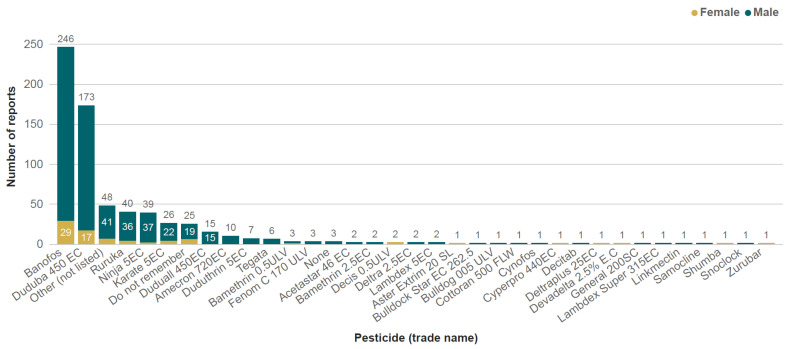
Frequency of pesticide products’ active ingredients reported by respondents experiencing acute pesticide poisoning symptoms in the last 12 months. Disaggregated by gender. Respondents could report up to three pesticide products by trade name for incidents involving single products or mixtures.

In total, 48% of the respondents reported experiencing acute health impacts within 24 h of pesticide exposure in the last 12 months. The proportion of men affected (51%) was higher than that of women (35%). The most frequently reported pesticide product associated with UAPP in the last 12 months was Banofos in 48% of cases, followed by Duduba 450EC, a pesticide co-formulation containing 350 g/L of chlorpyrifos and 100 g/L of cypermethrin (33% of cases; Figure 5). However, there are three different formulations of Banofos registered in Tanzania, namely Banofos 720EC (profenofos 720 g/L; Table S1), Banofos Super 520EC (profenofos 500 g/L + emamectin benzoate 20 g/L), and Banofos plus (profenofos 40%). During the interviews, respondents simply reported exposure to ‘Banofos’. However, when reporting the most recent incident involving Banofos only (*n* = 203), 36% reported exposure to a 720 g/L concentration, which we can assume was Banofos 720EC, 9% reported 500 or 20 g/L, which was likely Banofos Super 520EC, and 1% reported 40 g/mL, which was likely Banofos plus. A further 53% reported concentrations that we could not confidently assign, e.g., ‘0’ or ‘00’, accompanied by comments such as ‘He doesn’t remember’. Because all registered Banofos product formulations contain profenofos, we can confidently assume that profenofos was involved in all Banofos-related incidents, and thus, we have only included this active ingredient in our analysis of such incidents.

Profenofos was also the most frequently reported pesticide associated with the most recent incident of UAPP with a single pesticide (43% of cases), followed by the cypermethrin + chlorpyrifos co-formulation (Duduba 450EC; 26%), lambda-cyhalothrin (Ninja 5EC; 5% and Karate 5EC; 4%). However, 24 respondents (5%) were unable to recall the name of the pesticide. In addition, 31 respondents reported that the most recent incident followed exposure to a mixture of pesticides. Of these, the cypermethrin + chlorpyrifos co-formulation (Duduba 450EC) was the most frequently reported pesticide within the mixtures in 33% of the cases. Profenofos was the second most frequently reported pesticide within the mixtures (Banofos; 21% of cases).

When asked about their activity at the time of their most recent poisoning, most respondents (93%) reported that this occurred after applying pesticides to a crop (Table 2). Five respondents reported ‘Other (please specify)’, with two reporting that pesticide exposure occurred during the cleaning of clothes and equipment, one reported that this was due to pesticides stored in the bedroom, and one reported UAPP after transporting pesticides. One respondent did not provide this information.

The results of binary logistic regression revealed that UAPP was significantly influenced by gender and access to training (Table 3). Men had an approximately 65% higher likelihood of experiencing UAPP than women, with 51% of men reporting UAPP, compared to 35% of women. Farmers who did not receive PPE training had an approximately 30% higher likelihood of UAPP than their trained counterparts. However, a high proportion of those who had received PPE training still reported experiencing UAPP (43%), albeit lower than the 50% of those who had not received PPE training. Other factors investigated did not significantly affect the likelihood of UAPP.

### 3.4. Symptoms Reported

For participants who reported experiencing UAPP, 476 individuals reported their symptoms related to the most recent incident of acute pesticide poisoning with a single pesticide. Seven respondents declined to answer more detailed questions on the symptoms experienced. Based on the symptoms reported, the majority of cases were categorised as mild (71%), 20% moderate, and 9% severe. For incidents associated with profenofos, 145 (71%) were mild, 42 (20%) were moderate, and 18 (9%) were severe. Comparably, incidents associated with chlorpyrifos + cypermethrin co-formulations corresponded to a slightly higher proportion of severe symptoms, with 89 cases (67%) categorised as mild, 29 (22%) as moderate, and 14 (11%) as severe. These severe incidents were associated with Duduba 450 EC. For symptoms relating to incidents associated with lambda-cyhalothrin, 36 (80%) were mild, 6 (13%) were moderate, and 3 (7%) were severe; both Ninja 5EC and Karate 5EC were associated with these severe incidents. See Table 4 for the frequency of symptoms reported for different body systems.

**Figure 6:**
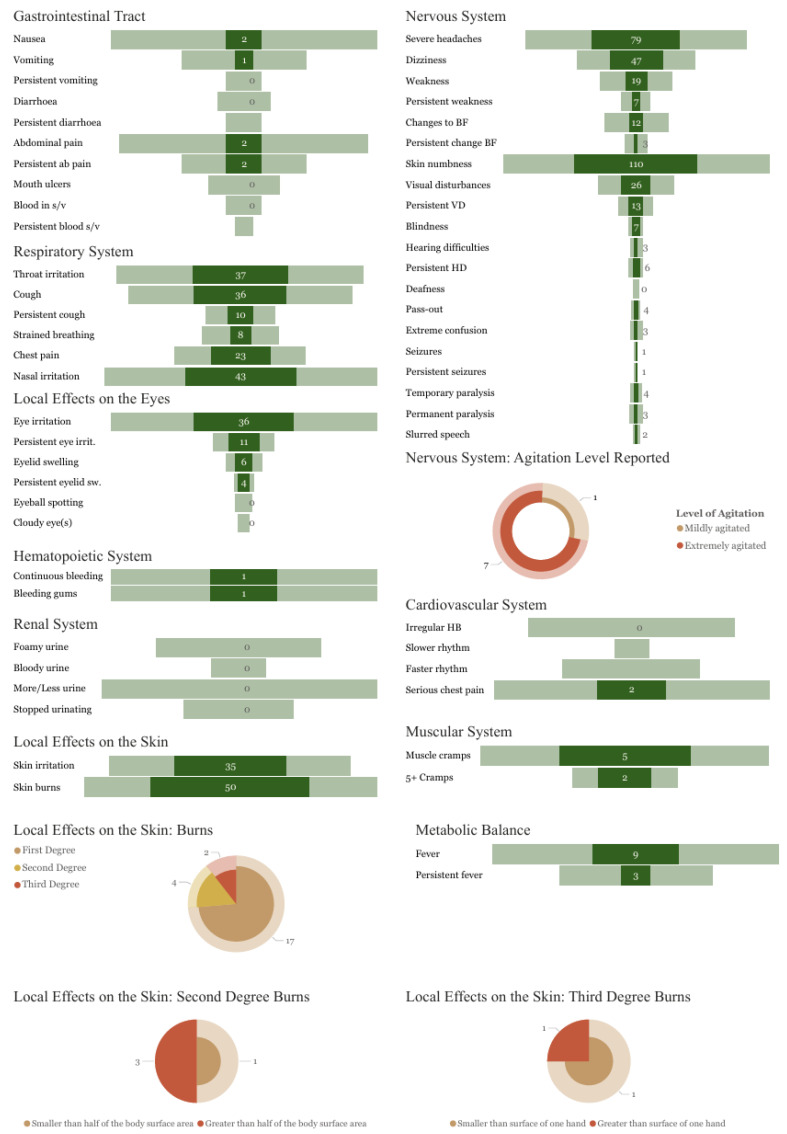
Frequency of symptoms reported for incidents involving profenofos (dark shaded) as a proportion of all reports (light shaded). ‘Persistent’ relates to symptoms lasting 48 h or longer; ab pain = abdominal pain; s/v = stools/vomit; chest pain = sharp stabbing pain in your chest which feels worse when you cough; eye irit. = eye irritation; eyelid sw. = eyelid swelling; cloudy eye(s) = when the eye is white, even where it is normally coloured; weakness includes any of the following symptoms: slowness or weakness when carrying out routine tasks and difficulty in walking or with balance; changes to BF relate to any of the following: increased or decreased salivation, decreased sweating, or difficulty in urinating; VDs = visual disturbances; HDs = hearing difficulties; HB = heartbeat.

Following exposure to profenofos, the most frequently reported symptoms (*n* = 205) were abnormal skin tingling or numbness (54%), severe headache (39%), visual disturbances (13%), muscle weakness, and abnormal involuntary movements (9%; Figure 6). A smaller number of more severe nervous system effects were reported, such as extreme agitation (3%), loss of consciousness (2%), temporary paralysis (2%), or permanent paralysis of a limb or specific body part (1%). Symptoms affecting other body systems included skin burns (24%; first degree defined as “red, painful, but no blisters”, 8%; second degree, defined as “red, blistered and swollen”, 2%; third degree defined as “white or brown and charred”, 1%), respiratory issues included throat irritation (18%), coughing (18%), and a sharp chest pain worse when coughing (11%). Further symptoms were reported by less than 5% of respondents.

**Figure 7:**
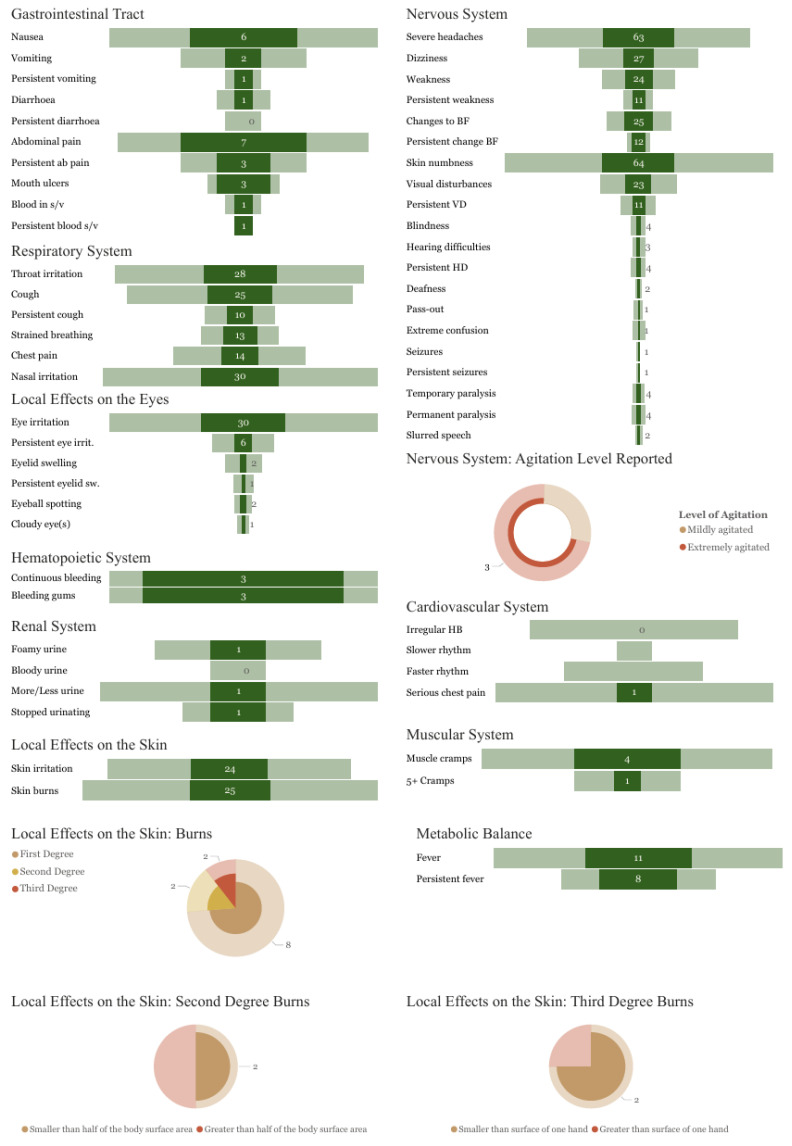
Frequency of symptoms reported for incidents involving the co-formulation of cypermethrin + chlorpyrifos (dark shaded) as a proportion of all reports (light shaded). ‘Persistent’ relates to symptoms lasting 48 h or longer; ab pain = abdominal pain; s/v = stools/vomit; chest pain = sharp stabbing pain in your chest which feels worse when you cough; eye irit. = eye irritation; eyelid sw. = eyelid swelling; cloudy eye(s) = when the eye is white, even where it is normally coloured; weakness includes any of the following symptoms: slowness or weakness when carrying out routine tasks and difficulty in walking or with balance; changes to BF relate to any of the following: increased or decreased salivation, decreased sweating, or difficulty in urinating; VDs = visual disturbances; HDs = hearing difficulties; HB = heartbeat.

Following exposure to chlorpyrifos + cypermethrin co-formulations (*n* = 132), the reported symptoms included abnormal skin tingling or numbness (48%), severe headaches (48%), muscle weakness, tremors, or abnormal movements (20%), increased or decreased bodily functions of salivation or sweating, difficulty urinating or constipation (19%), visual disturbances (17%), permanent paralysis of a limb or body part (3%), and extreme agitation and involuntary movements (2%; Figure 7). Additional symptoms were reported by less than 5% of respondents.

Symptoms reported by respondents in relation to lambda-cyhalothrin (*n* = 45) included abnormal skin tingling or numbness (49%), severe headaches (39%), dizziness (24%), increased or decreased bodily functions of salivation or sweating, difficulty urinating or constipation (16%), visual disturbances (11%) which were persistent (7%), and extreme confusion (7%). Additional symptoms reported included eye irritation (20%) and swelling of the eyelid (7%), as well as skin burns (18%), skin irritation (13%), and abdominal pain (7%). Further symptoms were reported by less than 5% of respondents.

Of those that reported single-product UAPP and consented to detailed questions on the most recent incident (*n* = 477), only 59 respondents (12%) reported seeking treatment in relation to the symptoms. Of these, 31 reported self-medication (6% of single-product UAPP incidents). The most frequently visited destination for treatment was the hospital by 24 individuals (5% of single-product UAPP incidents), and two individuals reported seeking treatment from health practitioners. Clinic and traditional healers were each reportedly used by one respondent.

Of the respondents reporting mild symptoms, 2% reported receiving treatment in formal healthcare settings (i.e., hospital: 3; health practitioner: 2; clinic: 1), and 5% reported informal treatment (self-medication: 17). For those reporting moderate symptoms, 8% reported receiving treatment in a formal healthcare setting (hospital: 8) and 12% reported informal treatment (self-medication: 10; traditional healer: 1). For those reporting severe symptoms, 30% reported receiving treatment in a formal healthcare setting (hospital: 13) and 9% reported informal treatment (self-medication: 4). Half (50%) of the individuals who visited a hospital reported that pesticides were mentioned on their diagnosis.

Similar incidents were reported to have occurred between one and eleven times in the past 12 months (Table 5). Half of respondents (50%) reported one occurrence, 26% reported two occurrences, and 15% reported three occurrences. A higher proportion of women reported only one occurrence (58%) compared to men (48%). Work days missed were reported by 73 individuals reporting UAPP, ranging from 1 to 60 days missed in the past 12 months, totalling 254 days missed.

## 4. Discussion

The results of this survey indicate a high rate of UAPP within smallholder cotton farmers and farm workers in Tanzania. Of 1074 respondents, 48% reported ill health effects within 24 h of exposure to pesticides in the past 12 months. The yearly incidence of UAPP is consistent with other assessments carried out in Tanzania [19], for cotton [7], and at a global level [5,27]. As with previous UAPP studies, the majority of incidents (80% of the most recent UAPP incidents with a single pesticide, 94% of mixtures) were related to a small number of active ingredients, namely profenofos (Banofos 720EC, Banofos Super 520EC, Banofos plus, and Amecron 720EC), lambda cyhalothrin (Ninja 5EC, Karate 5EC, Duduthrin 5EC, and Lambdex 4EC), and co-formulations containing both chlorpyrifos and cypermethrin (Duduba 450EC and Duduall 450EC). All of these active ingredients are considered highly hazardous pesticides (HHPs) according to the PAN HHP list [36] and, based on the survey results, all would fulfil criterion 8 of the FAO/WHO definition for HHPs [37].

### 4.1. Pesticides of Concern

The most commonly reported active ingredient involved in an incident of UAPP was profenofos, an organophosphorus (OP) insecticide, accounting for 39% of the most recent UAPP incidents reported. Profenofos is classified as WHO hazard class II and is banned in over 34 countries due to its negative impact on human health and the environment [38], yet it is one of the most commonly used pesticides in Africa [39] and is widely applied to cotton crops across the world [40]. Profenofos is an acetylcholinesterase (AChE) inhibitor that causes adverse effects on the central nervous and respiratory systems [41,42]. Mild symptoms of profenofos poisoning can include nervous system effects such as headaches, dizziness, nausea, and visual disturbances, as well as irritation to the eyes and respiratory system [43,44], with more severe OP poisoning resulting in bradycardia, bronchorrhea, hypotension, muscle weakness, confusion, and respiratory failure [45]. These reported symptoms are comparable to those reported by 203 respondents in this study following profenofos exposure. These included effects on the nervous system, such as severe headaches, visual disturbances, muscle weakness, extreme agitation, and loss of consciousness, and effects on the respiratory system, including reports of a persistent, painful cough and strained breathing. Further symptoms reported included skin irritation and chemical burns.

Symptoms of UAPP following exposure to co-formulations of cypermethrin and chlorpyrifos were frequently reported (28% of the most recent UAPP with a single product). Cypermethrin is a synthetic type II pyrethroid insecticide that is banned in more than 28 countries [38]; it is an endocrine disruptor and highly toxic to mammals, birds, fish, aquatic invertebrates, and honeybees. Chlorpyrifos is an OP insecticide banned in at least 39 countries [38] that is highly toxic to mammals, birds, fish, aquatic invertebrates, and honeybees. Furthermore, chlorpyrifos is currently being considered for listing under both the Stockholm and Rotterdam conventions due to its harmful effects on human health and the environment [46].

The effects of mixtures of pesticides are an understudied area, especially in Africa [47]. However, cypermethrin and chlorpyrifos are sold as co-formulations due to a reported synergistic effect, especially against resistant pests [48], which was identified as a result of their historical and widespread use in Africa as individual pesticides [39]. While the toxicity of each pesticide results in acute symptoms that impact various human body systems, notably the central nervous system [41,49], the interaction between OP and pyrethroids in co-formulations can also result in synergistic effects on humans [50]. The enzyme inhibition by OPs reduces the metabolism of pyrethroids, slowing down the process of the de-toxification of pyrethroids to non-toxic metabolites [49,50]. These synergistic effects result in more severe poisoning episodes, especially in comparison to incidents exclusively involving pyrethroids [51,52]. Although Wu, Chang, Chen, Tsai, Lee, Wang, Lee, Chen, Liu and Weng [52] found no increase in mortality or length of hospital stay, they identified an increase in respiratory failure. These findings were reflected in another study by Iyyadurai, et al. [53], who reported fewer ventilator-free days for patients with mixed cypermethrin and chlorpyrifos poisoning. However, they identified mortality in the group with mixed OP and pyrethroid poisoning, where no deaths occurred in the individuals poisoned by the single active ingredients.

Poisoning symptoms expected from exposure to chlorpyrifos are similar to those expected from exposure to other OPs such as profenofos, as listed above. Poisoning symptoms from mild–moderate exposure to synthetic pyrethroids include dermal irritations, increased sweating, mild neurological symptoms, and gastrointestinal symptoms. Increasingly severe cases can result in more serious neurological effects such as blurred vision and convulsions [54,55,56]. The symptoms reported by 132 respondents in this survey included nasal, throat, and skin irritation, persistent and painful coughing, and stabbing chest pain when coughing. Nervous system effects reported include severe headache, muscle weakness, abnormal skin tingling or numbness, visual disturbances, temporary or permanent paralysis in a specific limb, slurred speech, extreme agitation, and seizures.

Another synthetic pyrethroid, lambda cyhalothrin, was reported by 45% of respondents in this survey reporting UAPP. Lambda cyhalothrin is of specific concern for human health due to a high mammalian toxicity and a CLP classification of H330 (fatal if inhaled). Exposure to lambda cyhalothrin can result in mild acute symptoms such as irritation of skin, eyes, and the respiratory system, headaches, dizziness, nausea, fever, paraesthesia, muscle aches, and fatigue [50]. In cases of severe poisoning, symptoms can include convulsions, loss of consciousness, and acute lung injury [57]. The symptoms reported in the literature align with those reported by respondents in this survey, with a high proportion reporting abnormal skin tingling or numbness, severe headache, dizziness, eye, nasal, and throat irritation, sharp painful cough, visual disturbances, confusion, and agitation. Lambda cyhalothrin has also been identified in various studies as a potential endocrine disruptor [58,59].

A study in Tanzania, looking at coffee and vegetable production in Arumeru district, Arusha region, found a high proportion of poisoning incidents associated with lambda cyhalothrin, which highlights the impacts of lambda cyhalothrin beyond the cotton-producing communities in this study [20]. Other cases of UAPP from lambda cyhalothrin have been reported worldwide; in 2023, in the USA, through the EPA’s Incident Data System (IDS), 21 incidents were reported, with two classified as ‘major’ [60]. In 2017, Canadian policymakers undertook a re-evaluation of lambda cyhalothrin, identifying 95 incidents involving individuals during use or re-entry to recently sprayed areas [61]. In 2016, a study of 497 farmers and farm workers in Georgia found that 25% of the 61 incidents of UAPP reported were associated with lambda cyhalothrin. Subsequently, in 2017, Georgia submitted a notification to the Rotterdam Convention proposing the classification of two formulations of lambda cyhalothrin (50 g/L of EC and 50 g/L of CS) as Severely Hazardous Pesticide Formulations (document UNEP/FAO/RC/CRC.13/16). The two most common formulations reported in this Georgian study were Ninja 5EC and Karate 5EC, also 50 g/L of EC, providing further evidence of harms caused by such lambda cyhalothrin formulations.

A final concerning finding in this study was a high proportion of respondents who could not identify the pesticide that was associated with adverse health effects in the most recent incident (14%). This is concerning, as it implies a poor level of understanding of the pesticides being used. Although a degree of recall error is expected in self-reported studies [62], the high number of reports for unknown pesticides could be symptomatic of end users lacking access to the label information. This could be due to low literacy levels, which were not assessed in this study, labels being in foreign languages, or being damaged, resulting in being repackaged into unlabelled containers [20]. Although the majority of pesticides were obtained through legitimate routes, such as extension services, with most respondents reporting pesticides in their original containers, with original labels, the reliance on information printed on labels to prevent harms caused by pesticides leads to a narrative of ‘blaming the farmers’ for misuse even when there are systemic, socio-economic, cultural, and potential climatic barriers to safe use following label instructions [20,63,64].

### 4.2. Health and Economic Consequences of UAPP

In this study, it was found that only 12% of those reporting UAPP following exposure to a single product received treatment for their symptoms (59 out of 477 reports). Of those that received any kind of treatment for UAPP (*n* = 59), 53% self-medicated, while 41% received hospital treatment. However, only 6% of all respondents who reported single-product UAPP (*n* = 477) sought treatment from a formal healthcare setting (hospital, clinic, and health practitioner). Of the participants affected by UAPP, 29% reported moderate or severe symptoms. However, only 8% and 30% of those with moderate and severe symptoms, respectively, reported receiving treatment in a formal healthcare setting, further highlighting the underreporting of UAPP incidents. Another study in Tanzania, Lekei, Ngowi and London [19] also found low numbers of incidents reported to medical facilities. An associated study on the knowledge and practices of Tanzanian farmers identified multiple reasons for underreporting, such as poisoning symptoms being of mild severity and accepted as inherent in farming practices, high costs of medical treatment, long distances and poor access to healthcare facilities, and low expectations of care available, such as difficulties in diagnosis, reservations around treatment, and access to appropriate medical services or drugs [20]. Therefore, relying on medical records to estimate the level of UAPP is likely to be inaccurate and severely underestimate the true scale of the problem [19].

There are further concerns around the knowledge of healthcare providers to accurately identify and treat pesticide poisoning [65]. Less than half of those respondents who did interact with the formal healthcare system had pesticide poisoning recorded in their diagnoses. These factors emphasise the importance of surveillance systems to monitor pesticide poisoning, which would include engagement, knowledge sharing, and training with medical facilities and their workers.

Collectively, respondents reported missing a total of 254 days of work as a result of UAPP. Days missed were reported by 14% of individuals reporting UAPP, ranging from 1 to 60 days missed. This amounts to 49 days missed per year for every 100 farmers reporting UAPP. Days missed from work are symptomatic of a severe negative impact on worker health but also highlight the potential for a significant loss of household income, as Maumbe and Swinton [66] found amongst cotton farmers in Zimbabwe. Nationally, UAPP can also present economic burdens [19], with estimates putting the cost over USD 1 billion per year in the US alone [67]. A UNEP study looking at 37 Sub-Saharan countries estimated the cost of lost work days, outpatient medical treatment, and inpatient hospitalisation due to pesticide poisoning at USD 4.4 billion in 2005 [13]. In the Philippines, a study modelling farm productivity with pesticide use found that reductions in insecticide use improved net productivity, with authors concluding that improved productivity could be attributed to improved worker health [68]. The current study further supports the evidence for negative externalities that can arise from pesticide use, with implications for worker health affecting productivity and increasing the economic burden nationally and on households.

### 4.3. Conditions of Use

Our results revealed that there is a higher likelihood of UAPP amongst men than women. This may reflect the division of labour on farms in Tanzania, where men are more likely to apply pesticides to crops [69,70]. However, this does not acknowledge the higher vulnerability of women to certain health impacts from pesticide exposure [71,72] and the different routes to exposure they experience through other tasks on the farm [69]. For example, in our study, we found that all six of the reports for UAPP following entering a field recently treated with pesticides were by women. This suggests, perhaps, that women lack information about the spraying activity and/or the risks associated with bystander exposure. We also found reports of ‘other’ exposure routes, with two instances of reports of poisoning while cleaning the husband’s clothing and equipment. The different exposure routes experienced by women were recently highlighted in a study by Asmare, Freyer and Bingen [71]. Understanding women’s exposure routes is an important consideration for UAPP due to the often hidden nature of women’s role in agriculture [69].

Current regulatory mechanisms for the approval of HHPs rely heavily on the appropriate use of PPE, communicated through labels, to mitigate the risks of handling and applying highly toxic pesticides [73]. The risk mitigation measures proposed by PPE allow for the approval of hazardous chemicals in regions where barriers to access and the appropriate use of PPE are outside the control of farmers. For example, full PPE in hot weather can pose significant health risks [74], smallholder farmers may not be able to afford full PPE, and there may not be adequate availability in the local market [73]. Despite farmers facing such barriers, training is frequently identified as central to increasing PPE use amongst smallholder farmers. In this survey, those who had not received training were 30% more likely to be poisoned by pesticides than those who had received training. However, the increased use of PPE did not significantly reduce the likelihood of UAPP, with a high yearly incidence of UAPP (43%) still reported by individuals who had received training.

Garrigou, Laurent, Berthet, Colosio, Jas, Daubas-Letourneux, Jackson Filho, Jouzel, Samuel, Baldi, Lebailly, Galey, Goutille and Judon [73] have articulated serious concerns about the effectiveness of PPE when worn under uncontrolled circumstances, even if all the barriers to accessing PPE are overcome. Women face additional challenges to accessing effective PPE due to poor-fitting equipment that is designed for men, cultural expectations around dress, and traditional gender role divisions of labour [70,71]. In these circumstances, the assumption that the risks to human health caused by hazardous pesticides can be sufficiently mitigated by simply recommending PPE are wholly unjustified. The evidence indicates that, while training farmers and providing them with the recommended PPE will likely have some beneficial impact on reducing the levels of UAPP, it will not deliver the required level of protection and it will, of course, have no impact on bystander exposure or environmental impact.

It is notable that in the current study, chemical-resistant boots were reported as the most frequently reported item of PPE. Boots alone offer wholly inadequate protection, a finding reflected in another study in Tanzania [20]. Similarly to this current study, they also identified a high proportion of farmers reporting dust masks being used rather than appropriate respirators. The authors suggest that, ineffective against pesticides, dust masks may mistakenly increase confidence in protection and reduce other risk-mitigating behaviour.

Two of the top three most common forms of clothing reported in this study were ‘usual clothes’ and ‘ordinary clothes for pesticide spraying’. In addition to offering little protection, the use of already contaminated clothing during application presents an additional source of dermal exposure. The hand washing of contaminated clothing is also a potential exposure route, a task traditionally undertaken by women [70]. Even after cleaning, pesticide residue can persist in clothing [75], and inert ingredients in pesticide formulations can reduce the effectiveness of cleaning [76]. Exposure to pesticides via contaminated clothing emerged in this study, with multiple reports of UAPP following the washing of clothes.

### 4.4. Pests Targeted by HHPs and Available Alternatives

The pests most frequently targeted with pesticides by the participating cotton farmers were lepidopteran larvae (54% of reports), such as American bollworm (*Helicoverpa armigera*), and cotton stainers (*Dysdercus* spp.; 49% of reports). In accordance with these findings, the most frequently reported pesticides associated with incidents of UAPP in this study were insecticides that are frequently promoted for use against these pests, i.e., profenofos, cypermethrin, chlorpyrifos, and lambda-cyhalothrin [77,78]. However, as well as causing harm to human health, these HHPs negatively affect ecosystem services essential for sustainable cotton production, such as pollination, nutrient cycling, and pest regulation [79]. Profenofos, cypermethrin, chlorpyrifos, and lambda-cyhalothrin are recognised as posing a high to very high risk to beneficial insects in cotton [80,81,82]. The repeated use of pyrethroids in particular (e.g., lambda cyhalothrin and cypermethrin) is considered to increase the risk of *H. armigera* resurgence and pest outbreaks in cotton, as well as insecticide resistance development to the point where the insecticide fails to provide satisfactory control [82].

Fortunately, when following the ecologically based principles of agroecology and Integrated Pest Management (IPM), there is an array of tools and techniques that offer safer and more sustainable alternatives to HHPs for cotton pest management [77,78,83,84,85,86,87]. These include cultural techniques (e.g., crop rotation, intercropping, trap crops, crop residue removal, and field sanitation), biological control, physical methods, varietal resistance and tolerance, the use of ‘non-synthetic’ chemicals (e.g., neem), and the use of less hazardous synthetic chemicals as a last resort. We provide examples of these in Table 6.

There are several examples of successful cultural techniques used to develop unfavourable conditions for cotton pests. A recent review by Chi, et al. [88] of cotton intercropping for pest and disease management distils experiences from several countries and identifies several mechanisms by which cotton pest management can be supported by intercropping. Intercropping tends to be implemented by small- and medium-scale cotton farmers. However, field border strips planted with alfalfa or other crops have been used in large-scale cotton systems in Australia [89]. Field hygiene after harvest is also very important for cotton bollworms and cotton stainers, which are often present in stalks and green and fallen bolls [77,90]. This involves the careful removal of crop stalks and other parts after harvest to prevent any pests present from surviving or reproducing in the following season. Another technique for cotton pest management includes ‘cotton topping’, also known as ‘plant training’, which induces plant defence through artificial injury by cutting the shoot tips of cotton plants. A review by Llandres, et al. [91] concluded that this is a promising technique, particularly for smallholders, which can be used as part of an IPM programme to significantly reduce insecticide use and to improve productivity in cotton farming.

One of the most important elements of IPM is to encourage and conserve the natural enemies of pests based on the agroecological principle of enhancing beneficial biological interactions [87]. One crucial step towards achieving this aim is to avoid both calendar-based spraying and broad-spectrum insecticides. A second step is to provide refugia for natural enemies by increasing habitat diversity [89]. Furthermore, the biological control of cotton pests may be enhanced by augmentative mass releases of artificially reared predators or parasitoids, such as *Trichogramma* spp. [92,93], or by attracting predatory insects into the crop. A good example of a method to attract predatory insects into the cotton crop foliage is the food spray method, with proven success in smallholder cotton farms in Benin and Ethiopia [83,84,94,95].

Biopesticides (also referred to as biological pesticides or microbial biocontrol agents) and botanical pesticides are also proven effective alternatives to HHPs in cotton, with numerous studies showing efficacy against *H. armigera* and cotton strainers [96,97]. Examples are preparations of the bacterium *Bacillus thuringiensis* (Bt) or the fungi *Beauveria bassiana* and *Metarhizium* sp. In Tanzania, commercial products of Bt (e.g., Ascopel^®^ and BN3^®^), *B. bassiana* (e.g., Biobassiana^®^), and *Metarhizium* sp. (e.g., Real Metarhizium^®^) are currently registered (https://bioprotectionportal.com). The extracts of the seed or leaves of neem (*Azadirachta indica*) are widely used in cotton-growing countries, and they are available in Tanzania in several commercial products and are effective against several insect pests of cotton.

Overall, the phase-out of HHPs for cotton pests requires a phase-in of a set of IPM methods that suit the local context, rather than simply trying to replace a chemical with a single alternative product or technique. The growing organic cotton sector in Tanzania provides a testament that producing cotton without pesticides and especially HHPs is indeed possible. Between the 2019/2020 season and the 2020/2021 season, the volume of certified organic cotton fibre volume increased by 85%, and by 2023, organic cotton accounted for over 15% of national cotton production [98]. The lessons learnt by Tanzanian organic cotton producers will be particularly useful. To support the agricultural community to build their knowledge and confidence in using alternative methods, farmer and extension agent awareness raising through practical training, demonstrations, and experience sharing will be crucial [99,100,101].

**Table 6:** Selected studies providing relevant examples of alternatives to HHPs for lepidopteran larvae and cotton stainer pest management for cotton.

Method	Example	Country	Reference
**Cultural control**	Cotton intercropped with maize had significantly less damage (0.5%) than monocropped cotton (5–9%). In the cotton–maize intercropping system, *H. armigera* preferred to lay eggs on the maize plants.	China	Chi, Zhang and Dong [88]
	Cotton–basil intercropping significantly reduced total pest infestation and led to a 50% reduced abundance of the pink bollworm (*Pectinophora gossypiella*) in comparison with non-intercropped plots.	Egypt	Schader, et al. [102]
	Intercropping cotton with sesame and the release of *Trichogramma. chilonis* adults alternated with neem provided significantly better control of spotted bollworm, *Earias vittella,* and pink bollworm, *Pectinophora gossypiella,* compared to the control.	India	Devi, et al. [103]
	Field experiments conducted over a 6-year period showed that the densities of *H. armigera*, *Earias* spp., and Sudan bollworm, *Diparopsis watersi,* were significantly lower in topped compared with non-topped cotton plots.	Mali	Renou, et al. [104]
**Biological control**	Application of a supplementary food spray product attracted beneficial insects and significantly reduced the number of pests (including *Helicoverpa* spp.) and increased cotton yields and profitability.	Ethiopia/Benin	Mensah, Vodouhe, Sanfillippo, Assogba and Monday [83]; Amera, Mensah and Belay [84]
**Biopesticdes**	Application of neem oil in the laboratory and the field resulted in a considerable reduction in the hatching of the eggs of *H. armigera.*	India	Patel, et al. [105]
	100% mortality of red cotton stainers was reported following treatment with *B. bassiana* isolates.	India	Moorthi, et al. [106]
	*B. bassiana* and *Metarhizium rileyi* were as effective as lambda-cyhalothrin or chlorpyrifos against cotton stainers, with no significant difference in seed cotton yield.	South Africa	Malinga and Laing [97]
	Treatment with *Nomuraea rileyi* led to 87% mortality of *H. armigera*.	South Africa	Hatting [107]
	Treatment with *B. thuringiensis* led to a 95–100% and 76% *H. armigera* mortality under laboratory and field conditions, respectively.	South Africa	Malinga and Laing [96]
**Integrated package**	CABI Plantwise guidance for *H. armigera* management in cotton in Tanzania recommends regular scouting for pests and using neem-based products a max. of 3 times (usually 2.5–3 litres/ha or 50–60 mL/20 litres of water, or 20–50 g of neem seed cake or powder/litre water) to control small 1st–2nd instars of larvae if the ratio is less than 1:2 (bollworm: beneficial organisms). Field sanitation involving the removal of cotton plant debris and ratoon cotton as soon as harvesting is over is also recommended.	Tanzania	Ndomba and Kitandu [90]
	CABI Plantwise guidance for *D. cingulatus* management in Tanzania includes regular monitoring, hand-picking, and destroying the bugs in small plots at the beginning of infestations or spraying fresh custard apple leaf extract or pyrethrum powder. Field sanitation involving the removal of cotton plant debris and ratoon cotton as soon as harvesting is over is also recommended.	Tanzania	Mussa [77]

### 4.5. Limitations

This survey was limited to farmers and farm workers only. Thus, we did not capture the effects of pesticide exposure to other members of the farming household who were also exposed to pesticides [108]. As well as pesticide exposure during application, exposure can occur via various routes such as deliberate or accidental consumption, pesticide drift, the preparation of pesticide mixtures, or the cleaning or wearing of pesticide-contaminated clothing [71]. We also acknowledge that retrospective studies are subject to recall bias. However, evidence suggests that using a 12-month recall period can lead to a fair degree of reliability [61]. For example, a study by Gabbe, et al. [109] on self-reported sports injuries showed that survey participants are able to recall the number of injuries and the body region affected with a high degree of accuracy over a 12-month recall period when a clear and context-specific definition of the injury/symptom is provided (as was provided in our questionnaire). Overall, the authors consider that recall bias over 12 months is likely to lead to a conservative estimate of the scale of injury.

## 5. Conclusions and Recommendations

Our study showed a high rate of unintentional acute pesticide poisoning (UAPP) in farmers and farm workers in Northwestern Tanzania, particularly in relation to certain organophosphorus and pyrethroid pesticides that are considered highly hazardous. Abnormal skin tingling or numbness was the most commonly reported acute poisoning symptom. Mild nervous system effects such as headaches, dizziness, nausea, visual disturbances, as well as irritation to the eyes and respiratory system, and more severe OP poisoning resulting in muscle weakness, partial paralysis, agitation, confusion, and seizures were also reported.

The yearly incidence of acute pesticide poisoning uncovered in this study is similar to findings from other studies in Tanzania, suggesting that UAPP is a widespread problem. Our study also revealed that very few of those who experienced poisoning sought treatment from medical professionals, and as a result, very few cases will have been captured by formal—mostly hospital-based—surveillance systems. This could be one factor behind the very high levels of underreporting of UAPP in Tanzania, which have been found by other studies.

While our study found poisoning cases from over 30 different products and 15 active ingredient formulations, a striking feature of our study was that over 80% of poisoning events were associated with just four active ingredients: profenofos, cypermethrin, chlorpyrifos, and lambda-cyhalothrin. Previous studies in Tanzania have identified these active ingredients as commonly involved in poisoning incidents. Less hazardous alternatives are available for all of these pesticides, and experience elsewhere has shown that targeted bans on HHPs can dramatically reduce pesticide self-poisoning without affecting agricultural productivity.

We therefore recommend the following:

1. Phase out highly hazardous pesticides and prioritise the elimination of the four pesticides most commonly involved in poisoning incidents in this study—namely profenofos, cypermethrin, chlorpyrifos, and lambda-cyhalothrin.
2. Make less hazardous alternatives available and support farmers to access them.
3. Enforce regulatory control measures to prevent exposure to banned or restricted pesticides.
4. Provide information and training to farmers and farm workers on pesticide hazards and on ways to reduce pesticide-related risks, including PPE and, more importantly, on alternative pest management strategies that are based on agroecological or IPM principles.
5. Scale up community self-surveillance to complement poison centre/hospital-based pesticide poisoning surveillance.
6. More research into the health effects of co-formulations is needed, particularly those containing pyrethroids and organophosphates.

## Figures and Tables

**Table 1:** Social descriptive characteristics of respondents.

			Gender	
		Total *n* (%)	Male *n* (%)	Female *n* (%)
**Gender**		1074 (100)	881 (82)	193 (18)
**Age**	14–18	8 (1)	7 (1)	1 (1)
	18–40	567 (53)	464 (53)	103 (53)
	40–60	407 (38)	331 (38)	76 (39)
	60+	84 (8)	71 (8)	13 (7)
	Missing information	8 (1)	8 (1)	0
**Occupation Type**	Work on family farm *	903 (85)	746 (85)	157 (81)
	Work on family farm and do hired work	54 (5)	48 (5)	6 (3)
	Only do hired work	111 (10)	81 (9)	30 (16)
	Missing information	6 (1)	6 (1)	0

* defined as the farm that is owned by the farmer or their family.

**Table 2:** Frequency and percentage of reports for the main activity when the most recent poisoning incident occurred.

Activity When Poisoned (*n* = 501)	Total *n* (%)	Male *n* (%)	Female *n* (%)
Applying pesticides to a crop	464 (93)	418 (95)	46 (73)
Mixing/loading the pesticide ready to use	23 (5)	17 (4)	6 (10)
Entering a field treated with pesticides	6 (1)	0	6 (10)
Other (please specify)	5 (1)	0	5 (8)
Using pesticides in the home	3 (1)	3 (1)	0

**Table 3:** Results of logistic regression analysis to investigate variables influencing the occurrence of unintentional acute pesticide poisoning over the previous 12 months.

Variable	Category	B	S.E.	Wald	df	Sig.	Exp(B)	95.0% C.I. for EXP(B)	
								Lower	Upper
PPE	0 items			2.594	3	0.459			
	1 item	0.442	0.345	1.639	1	0.200	1.555	0.791	3.058
	2–3 items	0.284	0.350	0.657	1	0.418	1.328	0.668	2.640
	>3 items	0.297	0.362	0.672	1	0.412	1.345	0.662	2.735
Occupation type	Work on family farm *			1.363	2	0.506			
	Work on family farm and do hired work	−0.191	0.206	0.862	1	0.353	0.826	0.551	1.237
	Only do hired work	0.032	0.339	0.009	1	0.925	1.032	0.532	2.005
Gender		0.503	0.166	9.179	1	0.002	1.654	1.194	2.290
Access to training		0.265	0.147	3.267	1	0.041	1.303	0.978	1.737
Age		−0.060	0.096	0.385	1	0.535	0.942	0.780	1.138
Farm size	<1 ha			3.785	3	0.286			
	1–5 ha	0.336	0.363	0.860	1	0.354	1.400	0.687	2.850
	5–15 ha	0.453	0.256	3.149	1	0.076	1.574	0.954	2.597
	15 ha	0.296	0.269	1.217	1	0.270	1.345	0.794	2.277
Pesticides sold in original container		−0.276	0.321	0.738	1	0.390	0.759	0.405	1.424
Constant		−1.590	0.729	4.763	1	0.029	0.204		

* defined as the farm that is owned by the farmer or their family.

**Table 4:** Frequency and percentage of responses to body systems reported to have been affected within 24 h of using pesticides.

Body System	Total *n* (%)	Male *n* (%)	Female *n* (%)
Nervous system	476 (100)	414 (100)	62 (100)
Hematopoietic system	476 (100)	414 (100)	62 (100)
Local effects on the skin	367 (77)	327 (79)	40 (65)
Respiratory system	132 (28)	109 (26)	23 (37)
Local effects on the eyes	109 (23)	100 (24)	9 (15)
Metabolic balance	52 (11)	44 (11)	8 (13)
Muscular system	21 (4)	21 (5)	0 (0)
Gastrointestinal system	20 (4)	16 (4)	4 (6)
Cardiovascular system	18 (4)	15 (4)	3 (5)
Renal system	10 (2)	9 (2)	1 (2)

**Table 5:** Frequency and percentage of the number of occurrences of acute pesticide poisoning symptoms in the last 12 months broken down by gender.

Number of Occurrences	Total *n* (%)	Male *n* (%)	Female *n* (%)
**1**	236 (50)	200 (48)	36 (58)
**2**	125 (26)	108 (26)	17 (27)
**3**	73 (15)	68 (16)	5 (8)
**4**	22 (5)	19 (5)	3 (4)
**5**	10 (2)	10 (2)	0
**6**	8 (2)	8 (2)	0
**8**	1 (0.2)	1 (0.2)	0
**11**	1 (0.2)	0	1 (2)

## Data Availability

The raw data supporting the conclusions of this article will be made available by the authors, without undue reservation.

## Supplementary Materials

The following supporting information can be downloaded at: https://www.mdpi.com/article/10.3390/toxics13040300/s1, Table S1: Pesticide trade names reported during the survey in Tanzania, associated active ingredients and their WHO hazard classification.
