# Supplementary material for: Assessment of Unintentional Acute Pesticide Poisoning (UAPP) Amongst Cotton Farmers in Tanzania
Source: Toxics. 2025 Apr 11;13(4):300. doi: 10.3390/toxics13040300 (PMC12031290; doi:10.3390/toxics13040300)
Supplement: Supplementary file 1 [file toxics-13-00300-s001.zip › toxics-3549776-supplementary.pdf]

Supplementary Material

Table S1. Pesticide trade names reported during the survey in Tanzania, associated active ingredients and their WHO hazard classification.

Pesticide Trade Name	Active Ingredient	Number reporting poisoning incident in the last 12 months	WHO Hazard classification [35]	PAN HHP Criterion [35]
Acetastar 46EC	Acetamiprid (61 g/L)	2	II	
Amecron 720EC	Profenofos (720 g/L)	10	II	Highly toxic to bees
Aster Extrim 20SL	Acetamiprid (150 g/L)	1	II	
	Cypermethrin (50 g/L)		II	Highly toxic to bees
Bamethrin 0.5ULV	Deltamethrin (5 g/L)	3	II	Highly toxic to bees GHS+ C2 & R2
Bamethrin 2.5EC	Deltamethrin (25 g/L)	2	II	Highly toxic to bees GHS+ C2 & R2
Banofos 720EC	Profenofos (720 g/L)	246	II	Highly toxic to bees
Banofos super 520EC	Profenofos (500 g/L)		II	Highly toxic to bees
	Emamectin benzoate (20 g/L)		II	Highly toxic to bees & aquatic organisms Very persistent
Banofos plus	Profenofos (40%)		II	Highly toxic to bees
Bulldock Star EC 262.5	Beta-Cyfluthrin (12.5g/L)	1	Ib	WHO1b
	Chlorpyrifos (250 g/L)		II	EU GHS repro (1A ,1B)
Bulldog 005ULV	Beta-Cyfluthrin	1	Ib	WHO1b
Cottoran 500FLW	Fluometuron (500 g/L)	1	U	
Cyperpro 440EC	Cypermethrin (40 g/L)	1	II	Highly toxic to bees
	Profenofos (400 g/L)		II	Highly toxic to bees
Decis 0.5ULV	Deltamethrin	2	II	Highly toxic to bees GHS+ C2 & R2
Decitab	Deltamethrin	1	II	Highly toxic to bees GHS+ C2 & R2
Deltra 2.5EC	Deltamethrin (25 g/L)	2	II	Highly toxic to bees GHS+ C2 & R2
Deltraplus 25EC	Deltamethrin (250 g/L)	1	II	Highly toxic to bees GHS+ C2 & R2
Devadelta 2.5% EC	Deltamethrin (25 g/L)	1	II	Highly toxic to bees GHS+ C2 & R2
Duduall 450EC	Cypermethrin (150 g/L)	15	II	Highly toxic to bees
	Chlorpyrifos (300 g/L)		II	EU GHS repro (1A ,1B)
Duduba 450EC	Cypermethrin (100 g/L)	173	II	Highly toxic to bees
	Chlorpyrifos (350 g/L)		II	EU GHS repro (1A ,1B)
Duduthrin 5EC	Lambda cyhalothrin (50 g/L)	7	II	Fatal if inhaled

				Highly toxic to bees<br>GHS+ C2 & R2
Fenom C 170ULV	Profenofos	3	II	Highly toxic to bees
	Cypermethrin	1	II	Highly toxic to bees
General 200SC	Imidacloprid	1	II	Highly toxic to bees
Karate 5EC	Lambda cyhalothrin (50 g/L)	26	II	Fatal if inhaled<br>Highly toxic to bees<br>GHS+ C2 & R2
Lambdex 5EC	Lambda cyhalothrin (50 g/L)	2	II	Fatal if inhaled<br>Highly toxic to bees<br>GHS+ C2 & R2
Lambdex Super 315EC	Lambda cyhalothrin	1	II	Fatal if inhaled<br>Highly toxic to bees<br>GHS+ C2 & R2
	Chlorpyrifos		II	EU GHS repro (1A ,1B)
Ninja 5EC	Lambda cyhalothrin (50 g/L)	39	II	Fatal if inhaled<br>Highly toxic to bees<br>GHS+ C2 & R2
Ruruka	Sulphur	40		
Tegata	Lambda cyhalothrin	6	II	Fatal if inhaled<br>Highly toxic to bees<br>GHS+ C2 & R2
	Thiamethoxam		II	Highly toxic to bees
